# Distribution of alleles, genotypes and haplotypes of the *CYP2B6* (*rs3745274;* rs2279343) and *CYP3A4* (rs2740574) genes in the Malian population: Implication for pharmacogenetics

**DOI:** 10.1097/MD.0000000000026614

**Published:** 2021-07-23

**Authors:** Yaya Kassogue, Brehima Diakite, Oumar Kassogue, Issa Konate, Kadidiatou Tamboura, Zoumana Diarra, Mamoudou Maiga, Hind Dehbi, Sellama Nadifi, Cheick Bougadari Traore, Bakarou Kamate, Sounkalo Dao, Seydou Doumbia, Guimogo Dolo

**Affiliations:** a Faculty of Medicine and Odonstomatology, University of Sciences, Techniques and Technologies of Bamako, Mali; b Center of Listening, Care, Animation and Counseling, Bamako, Mali.; c Cellular and Molecular Pathology Laboratory, Faculty of Medicine and Pharmacy of Casablanca, University Hassan II, Morocco.

**Keywords:** *CYP2B6* (A > G) rs2279343, *CYP2B6* (G > T) rs3745274, *CYP3A4* (C > T) rs2740574, genotype, Mali

## Abstract

Cytochrome P450 enzymes play a central role in the phase I biotransformation process of a wide range of compounds, including xenobiotics, drugs, hormones and vitamins. It is noteworthy that these enzymes are highly polymorphic and, depending on the genetic makeup, an individual may have impaired enzymatic activity. Therefore, the identification of genetic variants in these genes could facilitate the implementation of pharmacogenetic studies and genetic predisposition to multifactorial diseases. We have established the frequencies of *CYP2B6* (*rs3745274;* rs2279343) and *CYP3A4* (rs2740574) alleles and genotypes in 209 healthy Malian subjects using TaqMan drug metabolism genotyping assays for allelic discrimination. Allele frequencies were 37% for *CYP2B6 rs3745274*; 38% for *CYP2B6* rs2279343; and 75% for *CYP3A4* rs2740574 respectively. Overall, the frequencies observed in Mali are statistically comparable to those reported across Africa except North Africa. The major haplotypes in *CYP2B6 rs3745274* and *CYP2B6* rs2279343 were represented by GA (60.24%) followed by TG (35.36%). We noted a strong linkage disequilibrium between *CYP2B6 rs3745274* and *CYP2B6* rs2279343 with D’ = 0.91 and r^2^ = 0.9. The frequencies of the genotypic combinations were 43.5% (GT/AG), 37.3% (GG/AA) and 11.5% (TT/GG) in the combination of *CYP2B6-rs3745274* and *CYP2B6-*rs2279343; 26.8% (GT/CC), 25.4%, (GT/CT), 17.2% and GG/CT in the combination *CYP2B6-*rs3745274-*CYP3A4*-rs2740574; 26.8% (AG/CC), 23.9% (AA/CC), 19.1% (AG/CT), and 11% (AA/CT) in the combination *CYP2B6*-rs2279343-*CYP3A4-*rs2740574, respectively. The most common triple genotype was GT/AG/CC with 24.9%, followed by GG/AA/CC with 23.9%, GT/AG/CT with 16.7%, and GG/AA/CT with 10%. Our results provide new insights into the distribution of these pharmacogenetically relevant genes in the Malian population. Moreover, these data will be useful for studies of individual genetic variability to drugs and genetic predisposition to diseases.

## 1 Introduction

Cytochrome P450 (CYP450) enzymes are a superfamily of hemoproteins involved in phase I of the metabolism drugs and many other xenobiotics, thus playing a vital role in protecting the body against toxic derivatives from harmful compounds.^[1]^ In fact, the biotransformation reactions carried out by CYP450 allow the body to protect itself against the occurrence of cancers and the toxic effects of drugs or even other endogenous or exogenous substances such as hormones and vitamins, thus contributing substantially to the body's homeostasis^[2]^; In mammals, CYP450 is expressed with a much higher concentration in the liver, but also to a lesser extend in the small intestine, lung and the kidney.^[3,4]^ It has been reported that 90% of the metabolism process are driven by CYP450 1–4 families.^[5]^ In addition, CYP450 is known to be highly polymorphic and, depending on genetic background, these enzymes can affect the pharmacokinetics and pharmacodynamics of drugs at the individual level, thus explaining interindividual genetic differences in response to drugs.^[6–8]^ The human CYP450 superfamily consists of 18 families, 42 subfamilies with 59 active genes.^[9]^ Among these, the *CYP2B6* and *CYP3A4* families have been widely explored in genetic association studies for multifactorial diseases such as gout,^[10]^ acute myeloid leukemia,^[11]^ chronic obstructive pulmonary disease^[12]^ as well than in pharmacogenetic studies including antiretrovirals (nevirapine, efavirenz),^[13]^ antimalarial (artemisinin),^[14]^ anticancer (chronic myeloid leukemia)^[15]^ and anesthetics (propofol).^[16]^ The *CYP2B6* and *CYP3A4* genes are located on the long arms of chromosomes 19q13.2 and 7q22.1 respectively.^[17,18]^ Several single nucleotide polymorphisms (SNPs) have been identified in these genes at the exonic, intronic, and promoter regions. Some of these variants classified as loss-of-function greatly increase the plasma concentrations of substrates, while the others classified as a gain-of-function reduce plasma concentrations.^[19]^ It was reported that the replacement of G by T at position 516 of the CYP2B6 gene resulting in a Glutamine–Histidine change reduces significantly the catalytic activity of the gene. In addition, several studies have noted that the CYP2B6 rs3745274 homozygous mutant genotype is associated with increased plasma concentrations of efavirenz, leading to side effects related to the central nervous system.^[20,21]^ However, the CYP2B6 rs2279343 which leads to the change from Lysine to Arginine at position 262 induces a lower plasma exposure to efavirenz.^[22]^ The SNP rs2740574 in *CYP3A4* promoter increases the activity of *CYP3A4* and seems to be associated with the metabolism of tacrolimus requiring dose readjustments.^[23]^ It is important to stress that the allelic distribution of these genes varies from one population to another around the world, thus displaying strong interindividual, interethnic and geographical disparities.^[24]^ To the best of our knowledge, these data are missing for the Malian population. In addition, Mali is a tropical country where AIDS and malaria are major public health problems. It should be mentioned that most of the drugs used to treat these conditions are primarily metabolized by CYP450. For this reason and given the clinical importance of these genes encoding CYP450, we carried out the present study in order to establish the allelic and genotypic frequencies of *CYP2B6* in exons 4 and 5 (rs3745274, [Q172H]; rs2279343, [K252R]) and *CYP3A4* in the promoter (rs2740574) in our healthy population. The data from this study will be used to better design genetic association studies such as individual susceptibility to multifactorial diseases or variability in drug response.

## 2 Materials and methods

### 2.1 Study participants

The study protocol was approved by the Ethics Committee of the Faculty of Medicine and Odontostomatology (FMOS) /Faculty of Pharmacy (FAPH), University of Sciences, Techniques and Technologies of Bamako (USTTB) under the number 2018/113/CE/FMPOS. All participants received a detailed explanation of the study protocol before agreeing to sign the informed consent. For molecular biological purposes, five milliliters of peripheral venous blood were collected from each participant in a tube containing EDTA as an anticoagulant and stored at -20°C at the laboratory of the International Center of Excellence in Research-Mali (ICER, Mali). In sum, we recruited 209 healthy and unrelated subjects, including 120 female and 89 male from August 2018 to January 2019. The study participants recruited at the Department of Infectious and Tropical Diseases, University Hospital Center of Point G.

### 2.2 *CYP2B6* (rs3745274, rs2279343) and *CYP3A4* (rs2740574) genotyping

Genomic DNA was isolated from white blood cells using the Gentra Puregene Blood Kit according to the manufacturer's instructions. Reagents for TaqMan drug metabolism genotyping assays for allelic discrimination (Applied Biosystems Genotyping Assays) were used to establish the genetic profile of study participants. The following assays identification numbers were used: C_7817765_60 rs3745274 for *CYP2B6*, C_1837671_50 rs2740574 for *CYP3A4* rs2740574, a custom designed TaqMan assay was performed for *CYP2B6* rs2279343. The 7500 Fast Real-Time PCR System (Applied Biosystems) was used to genotype the different SNPs. PCR mixture consisted of a 5 μl TaqMan master mix (2X), 0.5 μl TaqMan drug metabolism genotyping assays mix (20X), and 1 μl of DNA completed to 10 μl with nuclease free water. The PCR run method was as follows: an initial step at 60°C for 30 s, hold stage at 95°C for 10 min followed by 40 cycles: step 1 at 95°C for 15 s and step 2 at 60°C for 1 min supplemented by a read stage at 60°C for 30 s.

### 2.3 Statistical analysis

The SPSS version 16 statistical package (SPSS Inc., Chicago, IL, USA) was used to assess the frequencies of *CYP2B6* rs3745274, rs2279343) and *CYP3A4 (*rs2740574) in our population. The chi-square (X^2^) test was used to check whether the distribution of alleles is in Hardy–Weinberg equilibrium or not. We compared the frequencies observed in Mali with the frequencies of other populations with the same test. Analysis of pairwise linkage disequilibrium between SNPs in *CYP2B6* was performed using SNPstats.^[25]^ A *P*-value less than .05 was considered statistically significant.

## 3 Results

**Figure 1:**
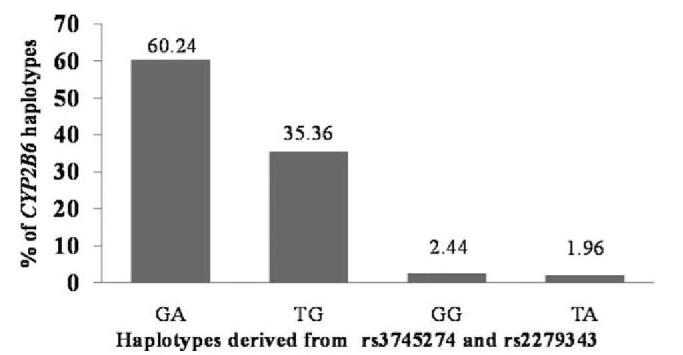
Haplotypes distribution of *CYP2B6*-rs3745274 and *CYP2B6*-rs2279343 in the Malian population.

**Figure 2:**
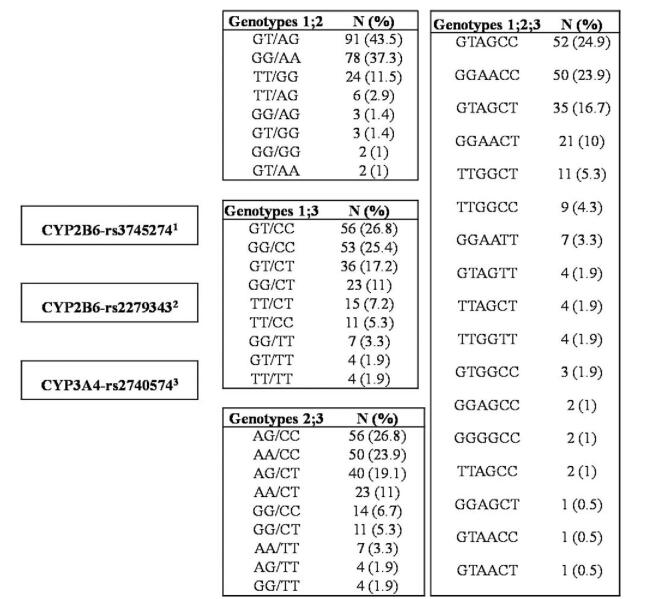
Distribution of combined genotypes between *CYP2B6*-rs3745274*, CYP2B6*-rs2279343 and *CYP3A4-*rs2740574.

In the present work, we established the allele and genotypic frequencies of the *CYP2B6* and *CYP3A4* genes in 209 healthy Malian subjects using the TaqMan genotyping method. Our study group was composed of 57.4% women and 42.6% men with a mean age of 30.5 ± 11.9 years (range 18–78 years). Table 1 displays the distribution of the three SNPs in the Malian population, The profiles of the genotypes observed in the *CYP2B6* gene were 40% (GG), 46% (GT), and 14% (TT) in exon 4; 38% (AA), 48% (AG), and 14% (GG) in exon 5, respectively. In *CYP3A4* gene, the genotypes observed were 57% for CC, 35% for CT and 7% for TT. The minor allele frequencies were 37%, 38%, and 25% for *CYP2B6*-rs3745274, *CYP2B6-*rs2279343 and *CYP3A4* rs2740574, respectively. We found no deviations from the Hardy–Weinberg equilibrium in the SNPs examined. The distribution of *CYP2B6*, and *CYP3A4* was statistically comparable between males and females. Major haplotypes identified from exons 4 and 5 in the *CYP2B6* gene were observed in subjects carrying GA (60.24%) and TG (35.36%), while minor haplotypes were found in individuals harboring GG with 2.44% or TA with 1.96% (Fig. 1). A strong linkage disequilibrium was observed between the two SNPs in *CYP2B6* with D’ = 0.91 and r^2^ = 0.9. The frequencies of the different genotype combinations are summarized in Figure 2. When analyzing the combinations of double genotype we noted in genotypes 1 and 2 (*CYP2B6*-rs3745274-*CYP2B6-*rs2279343), that 43.5% of the participants carried the heterozygous profile GT/AG, followed by the bearers of the homozygous wild-type profile GG/AA with 37.3% and 11.5% harboring the homozygous mutant TT/GG profile. The most frequent combinations in genotypes 1 and 3 (*CYP2B6*-rs3745274-*CYP3A4-*rs2740574) were represented by GT/CC (heterozygous/homozygous wild-type) with 26.8%, GG/CC (both homozygous wild-type) with 25.4%, GT/CT (both heterozygous) with 17.2% and GG/CT (homozygous wild-type/heterozygous) with 11%, respectively. Compared to this, a similar pattern of inheritance of the combined genotypes was observed in genotypes 2 and 3 (*CYP2B6-*rs2279343-*CYP3A4-*rs2740574) with 26.8% of subjects harboring AG/CC, 23.9% for AA/CC, 19.1% for AG/CT, and 11% for AA/CT. In terms of the combination of the three genotypes, we observed that 24.9% of our participants simultaneously inherited the GT/AG/CC genotypes, followed by GG/AA/CC with 23.9%, GT/AG/CT with 16.7%, and GG/AA/CT with 10%. Of the 209 participants, the triple genotype frequencies associated with impaired enzymatic activities were 5.3% for subjects harboring the TT/GG/CT profile, 4.3% for TT/GG/CC, and 1.9% for TT/GG/TT.

**Table 1:** Allelic and genotypic profiles of *CYP2B6*-rs3745274*, CYP2B6*-rs2279343 and *CYP3A4* rs2740574 in 209 Malian subjects.

Genes/SNPs	Genotypes/Alleles	All Participants, N (%)	Male, N (%)	Female, N (%)	*P*
*CYP2B6*^∗^6*/* rs3745274	GG	83 (40)	40 (45)	43 (36)	.32^∗^
	GT	96 (46)	39 (44)	57 (48)	
	TT	30 (14)	10 (11)	20 (17)	
	G	262 (63)	119 (67)	143 (60)	
	T	156 (37)	59 (33)	97 (40)	
	HWE: *P*	*.77*	*1*	*.85*	
*CYP2B6*^∗^4*/* rs2279343	AA	80 (38)	39 (44)	41 (34)	
	AG	100 (48)	40 (45)	60 (50)	.32^†^
	GG	29 (14)	10 (11)	19 (16)	
	A	260 (62)	118 (66)	142 (59)	
	G	158 (38)	60 (34)	98 (41)	
	HWE: *P*	*.88*	*1*	*.85*	
*CYP3A4/* rs2740574	CC	120 (57)	49 (55)	71 (59)	
	CT	74 (35)	33 (37)	41 (34)	
	TT	15 (7)	7 (8)	8 (7)	.83^‡^
	C	314 (75)	131 (74)	183 (76)	
	T	104 (25)	47 (26)	57 (24)	
	HWE: *P*	*.46*	*.78*	*.61*	

∗ X^2^ = 2.27.

† X^2^ = 2.3.

‡ X^2^ = 0.37.

## 4 Discussion

One of the major challenges of the twenty-first century for researchers and clinicians is to be able to predict the therapeutic outcomes of an individual for a given drug on the one hand and, on the other hand, to understand the development of multifactorial diseases. *CYP450* is well known to be involved in the metabolism of many drugs as well as in the development of multifactorial diseases. This is the first time that the distribution of *CYP2B6*-rs3745274, *CYP2B6-*rs2279343 and *CYP3A4-*rs2740574 has been established in Malian healthy subjects. The frequency of the *CYP2B6*-rs3745274 mutant T allele observed in the Malian population was 37%, which is comparable to those observed in Central Africa (Cameroon 37%),^[26]^ East Africa (Tanzania) 34%,^[26]^ South Africa 36%^[26]^ and in West African countries including Ivory Coast (38%),^[27]^ Nigeria (36.3%),^[28]^ and Ghana (46%).^[29,30]^ Looking at data from North Africa, we found that the frequency observed in Mali was statistically higher than that reported is Egypt (28.8%) *P* = .03,^[31]^ but lower than that observed in the Moroccan population (55.5%) *P* = .000.^[15]^ As for *CYP2B6-*rs2279343, the frequency of the mutant G allele was 38% in the Malian population. This frequency was almost comparable to the frequencies reported in other African countries such as Egypt 30.4%.^[31]^ Cameroon 35%,^[26]^ and South of Africa 36%^[26]^ (Table 2). The frequencies of *CYP2B6* in exons 4 and 5 reported in the European and Asian population were slightly lower than those observed in Mali.^[26]^ Overall, the minor allele frequencies for both *CYP2B6*-rs3745274 and *CYP2B6-*rs2279343 observed in Mali were similar to the frequencies reported in the 1000Genomes project for the African population.^[32]^ We noted a high LD between the two SNPs in *CYP2B6* gene, showing their proximity. The frequencies of haplotypes in the Malian population were comparable to those observed in Cameroon and South African.^[26]^ It has been reported that the *CYP2B6-*rs2279343 variant is associated with an increase protein expression with strong enzymatic activity, and that the opposite effect is observed with the *CYP2B6-*rs3745274 variant.^[31,33]^ In our analysis, we noticed in genotypic combination 1;2 that approximately 43.5% of our participants carried heterozygous genotypes while subjects harboring normal and mutated homozygous genotypes were 37.3% and 11.5%, respectively. These results could suggest a compensatory effect between these two SNPs which also exhibit strong linkage disequilibrium. The involvement of these SNPS of *CYP2B6* gene in the metabolism of various drugs has been demonstrated by several studies.^[14,34]^ Efavirenz and nevirapine (two non-nucleotide reverse transcriptase inhibitors) are among the flagship drugs whose metabolism is influenced by *CYP2B6* SNPs. In fact, the association between high plasma concentrations of drugs leading to side effects on the central nervous system and the presence of the *CYP2B6* rs3745274 polymorphism has been reported in HIV patients receiving efavirenz, while the *CYP2B6* rs2279343 polymorphism has been found to be associated with increased metabolic activities with low plasma exposure.^[22]^ Thus, the genotyping of these SNPs in our HIV-positive patients could allow better therapeutic management while limiting the occurrence of central nervous system related side effects. In addition, the drugs prescribed to treat cases of malaria are currently artemisinin-based combinations and these are metabolized by *CYP2B6* genes. Carriers of *CYP2B6*-rs3745274 variant have been reported to undergo a drug interaction when efavirenz and lumefantrine are co-administered, resulting in decreased plasma lumefantrine concentrations. AIDS and malaria are common in Mali; therefore, establishing the genetic profile of *CYP2B6* in our HIV patients infected with malaria parasite will facilitate better classification of patient according to their genotypes and alternative use of others antimalarials drugs. The frequency of *CYP3A4* rs2740574 alleles observed in the Malian population (75%) was comparable to those reported in Ghana 80.5%,^[35]^, Senegal 78.3%,^[35]^ Cameroon 78%,^[26]^ Tanzania (74%),^[29]^ South Africa (66%),^[26]^ but significantly higher than those reported in North African Countries, including Morocco (24.4%),^[36]^ Algeria (16.9%),^[37]^ Tunisia (10.8%),^[36]^ and Libya (19.9%)^[37]^ (Table 2). The frequency of *CYP3A4* rs2740574 is almost less than 5% in Europe and Asia, but more than 65% in populations of black African origin.^[32]^ The impact of *CYP3A4* rs2740574 polymorphism on gene expression remains controversial when we review the data in the literature. However, some authors have reported that this SNP influences the metabolism of antimalarial drugs such as artemisinin combination and quinine.^[29,30]^ It has been reported that due to the reduced binding of a transcriptional repressor, the *CYP3A4* rs2740574 variant leads to an increase in enzyme expression.^[38]^ However, a study in pregnant women from Tanzania reported that this variant was associated with decreased enzyme activity and that the clinical and parasitological therapeutic responses were comparable between homozygous wild-type and mutated carriers.^[39]^ Therefore, the identifying of the *CYP3A4* rs2740574 variant in our patients with malaria could allow us to better understand the pharmacogenetic impact of this gene in the Malian population and to identify later those who are at high risk of toxicity. Besides infectious diseases, these SNPs have been documented in their susceptibility to certain chronic diseases such as neoplastic diseases. Zhou et al, in a meta-analysis, showed that carriers of the *CYP3A4* rs2740574 variant in African populations are at increased risk of prostate cancer.^[40]^ Wang et al, in a meta-analysis showed that renal transplant recipients harboring *CYP3A4* rs2740574 variant require a lower dose of cyclosporine compared to those carrying wild type alleles of *CYP3A4*.^[41]^ For this reason, the results of this study, which already provide an overview of this polymorphism in the Malian healthy population, will better guide the evidence-based prescription of this drug.

**Table 2:** Distribution of *CYP2B6*-rs3745274, *CYP2B6*-rs2279343 and *CYP3A4*-rs2740574 in different African populations.

Populations	N	rs3745274**T** (%)	rs2279343**G** (%)	rs2740574**C** (%)	References
West Africa					
Malian (this study)	209	37	38	75	Present study
Ivory Coast	41	38	–	–	^[27]^
Nigeria	150	36.3	–	–	^[24]^
Ghana	80/118	48.8	47.5	80.5	^[35,42]^
Senegal	173	–	–	78.3	^[35]^
North Africa					
Egypt	120	28.8^∗^	30.4		^[43]^
Morocco	64	55.5^∗^	-	24.4^∗^	^[15,36]^
Algeria	83	–	–	16.9^∗^	^[37]^
Tunisia	102	–	–	10.8^∗^	^[36]^
Libya	93	–	–	19.9^∗^	^[37]^
Central Africa					
Cameroon	69	37	35	78	^[26]^
East Africa					
Tanzania	134/121	34	–	74	^[29]^
Ethiopia	264	31.4	–		^[44]^
South Africa					
South Africa	153	36	36	66	^[26]^
Zimbabwe	118	45	–	–	^[27]^

∗ statistically significant when compared to Malian.

## 5 Conclusion

In the present study, we identified the genetic profiles of *CYP2B6* rs3745274*, CYP2B6* rs2279343 and *CYP3A4* rs2740574 in a sample of the Malian population. The results obtained provide new insights on the distribution of drug metabolizing enzymes in the Malian population not described previously. In addition, they will contribute to the implementation of pharmacogenetic studies on drugs, which are substrates of these genes, in particular antiretrovirals and antimalarials for better genotype–phenotype correlation.

## Acknowledgments

The authors would like to express special thanks to the study participants; the university clinical research center (UCRC-Mali); Prof Cheick Fantamady Traore, Prof Mahamadou Diakite and Dr Mamadou Coulibaly for logistical support.

## Author contributions

**Conceptualization:** Yaya Kassogue, Brehima Diakite.

**Data curation:** Yaya Kassogue, Oumar Kassogue, Issa Konate, Kadidiatou Tamboura.

**Formal analysis:** Yaya Kassogue, Brehima Diakite.

**Funding acquisition:** Yaya Kassogue.

**Investigation:** Brehima Diakite, Konate Issa, Kadidiatou Tamboura, Zoumana Diarra, Mamoudou Maiga, Sounkalo Dao.

**Methodology:** Yaya Kassogue, Brehima Diakite, Oumar Kassogue, Guimogo Dolo.

**Supervision:** Hind Dehbi, Sellama Nadifi, Cheick Bougadari Traore, Bakarou Kamate, Sounkalo Dao, Seydou Doumbia, Guimogo Dolo.

**Validation:** Yaya Kassogue.

**Writing – original draft:** Yaya Kassogue.

**Writing – review & editing:** Yaya Kassogue, Brehima Diakite.
